# A Comparison Between Physical Methods Based on Mechanical Action and Pharmacotherapy in the Treatment of Discogenic Low Back Pain

**DOI:** 10.3390/healthcare13172238

**Published:** 2025-09-08

**Authors:** Julia Pingot, Michał Słupiński, Adam Lipski, Marta Woldańska-Okońska

**Affiliations:** 1 Department of Nursing, Faculty of Social Sciences, Piotrkowska Academy, 97-300 Piotrków Trybunalski, Poland; 2 Rehabilika-Centre for Rehabilitation and Adult Age Medicine, 93-029 Lodz, Poland; 3 Department of Internal Medicine, Rehabilitation and Physical Medicine, Medical University of Lodz, 90-419 Lodz, Poland; 4 Collegium Medicum, Społeczna Akademia Nauk University, 90-113 Lodz, Poland

**Keywords:** multiple impulse therapy (MIT), mechanical diagnosis and therapy (MDT), McKenzie, DLBP

## Abstract

**Background/Objectives**: Back pain affects a large number of people and, therefore, represents a significant financial burden for the state. In most cases, it can be treated conservatively. The aim of this study is to evaluate and compare the effects of multiple impulse therapy (MIT), the McKenzie method, axial traction using the Saunders lumbar lift, and NSAID pharmacotherapy in patients with discogenic low back pain (DLBP). **Methods**: All patients completed a subjective evaluation of pain, both before and immediately after treatment, providing values on the Laitinen and VAS scales. The Schober test was performed in all groups. Pain and mobility were also assessed 30 days after the completion of treatment. **Results**: In all groups of patients, a significant improvement was obtained both at the end of treatment and 30 days after the applied therapies. On the Laitinen scale, the best results were obtained with McKenzie therapy and were similar with Saunders traction. On the VAS scale, the best results were observed in the group of patients treated with multiple impulse therapy and according to the Schober test. **Conclusions**: Multiple impulse therapy functions as a valuable modality for pain control for treating patients with discogenic low back pain compared to McKenzie MDT and Saunders traction. MIT is well-tolerated by patients, completely safe, and non-invasive. Physiokinetic methods such as Saunders’ traction, McKenzie, and MIT showed greater analgesic efficacy when compared to drug treatment in patients with discogenic low back pain.

## 1. Introduction

Usually, back pain is not a serious condition and can be treated conservatively. However, it affects a large number of people and, therefore, represents a significant financial burden for the state, including the cost of medical procedures, reduced productivity at work, and absenteeism and early disability judgments, among other things [1]. In total, 11–12% of the population is disabled due to LBP. In addition, chronic pain, affecting 7–10% of the global population, often leads to comorbid conditions such as anxiety, insomnia, and depression [2].

Pain is the predominant symptom of musculoskeletal diseases in the back [1], with a significant proportion of cases progressing from acute to chronic pain. Current clinical care frequently adopts a structural–anatomical perspective, which neglects the pathophysiological processes responsible for pain [3]. Categorizing pain into three basic mechanisms—neuropathic pain, nociceptive pain and central sensitization—we consider chronic nociceptive disc-derived pain with etiology confirmed via the observation of MRI images in this study.

Given the significant incidence of chronic low back pain and its etiology, mechanical methods of action were used such that the comparison would involve a similar type of stimulus. Thus, a comparison was made between the relatively new and less-studied multiple impulse therapy (MIT) and the diagnostic and therapeutic McKenzie method and Saunders tractions. All of these methods have an effect on the tension of the peri-spinal tissues and are intended to cause a reduction in pain by reducing neuromuscular tension [4,5,6]. Further, we considered the age and gender of the subjects and the effect of movement on oxidative stress.

The intervertebral disc (IVD) is the largest non-vascular element in the human body and has a small number of cells. Hydrostatic pressure strongly affects the nucleus pulposus (NP). The cells of the fibrous ring and nucleus pulposus are exposed to high mechanical stress and are adapted to anaerobic metabolism at low oxygen pressure and acidic pH. Nevertheless, chronic oxygen and glucose deficiencies have deleterious effects on the viability of most cells; oxygen deficiency causes them to become inactive. Disc and herniated disc cells behave differently; they are still biologically active and produce a number of factors associated with inflammation, including nitric oxide, prostaglandin E2 (PGE2), ECM meta-protease, and pro-inflammatory cytokines, including tumor necrosis factor alpha (TNF-α), interleukin-1α (IL-1α), interleukin-1β (IL-1β), interleukin-6 (IL-6), and factors that stimulate the accumulation of granulocytes and macrophages. These factors exacerbate the inflammatory process and, consequently, pain [2]. Individuals with low back pain exhibit increased muscle activity (muscle tension) and worse postural recovery compared to the asymptomatic group; health restitution is negatively related to muscle activity, pain, and disability. Muscle activity is also correlated with psychological factors, which can affect the return to normal posture through their influence on muscle activity. The results of the study in [7] confirm the importance of muscle activity in LBP, particularly as a factor through which psychological stimuli can affect the clinical outcome. The mediating role of muscle activity in the correlation of psychological factors and pain suggests that interventions that are able to reduce muscle tension may be particularly beneficial for patients exhibiting such characteristics, which may help to guide LBP treatments [7]. Accordingly, a measure of spinal mobility in the form of the Schober test was included in this study.

The hypothesis of the analgesic efficacy of MIT’s multiple impulse therapy for lumbar spine pain syndromes is as follows: normalizing the tension of the paraspinal muscles in patients suffering from this condition has an analgesic effect, compared to the McKenzie diagnostic and therapeutic method, axial traction of the spine with the Saunders lumbar traction, and the effectiveness of treatment with non-steroidal anti-inflammatory drugs (NSAIDs).

The aim of this study is to evaluate and compare the effects of multiple impulse therapy (MIT), the McKenzie method, axial traction using the Saunders lumbar traction, and NSAID pharmacotherapy in patients with discogenic low back pain.

## 2. Materials and Methods

This study was performed in 4 groups of 70 patients each, aged 30–60 years, including both sexes. All patients were treated at the Motor Rehabilitation Center in Piotrkow Trybunalski (now FiortClinic) for low back pain in the course of degenerative disease, diagnosed as discogenic pain, which was confirmed via imaging diagnostic tests.

The first group of subjects underwent axial spinal traction in the supine position using a Saunders lumbar lift. The duration of the treatment ranged from 5 to 12 min, with a force of ½ the patient’s body weight. The treatments were performed twice a day for 15 days.

An intervention was provided to the second study group with the McKenzie diagnostic and therapeutic method. Here, the overpronation pattern was used, acting dynamically without a lateral component. Patients performed the overstretch pattern in 5–6 series per day of 12 to 15 repetitions, depending on their history, for 15 days.

Another group was treated with multiple impulse therapy using the PulStarFRAS diagnostic and therapeutic device. In all patients, diagnostics were performed along with the treatment. Between 2 and 5 treatments were implemented, with an average of two treatments per week.

The last comparison group was patients treated only with a pharmacological NSAID: ketoprofen, 2 times a day at 100 mg.

### 2.1. Inclusion Criteria

- Informed consent to the treatments;
- Chronic low back pain syndrome of discogenic origin, lasting at least 12 weeks;
- Age between 30 and 60 years;
- No contraindications to the method.

### 2.2. Exclusion Criteria

- Tumors of the spinal cord, roots, and meninges, or primary and metastatic tumors of vertebral neoplasms;
- Tuberculosis of the spine;
- Fresh fractures;
- Significant hypermobility of the spine;
- Severe form of osteoporosis;
- Individuals after spinal surgery.

All patients received physical treatment using a Sollux lamp once a day with a blue filter, lasting 12 min, to ensure that the conditions of the study were the same in all groups.

All patients self-reported their subjective assessment of pain, giving values on the Laitinen and VAS scales, before starting and immediately after completing the multiple impulse therapy [5,8], McKenzie MDT [9,10], Saunders traction therapy [11], or drug treatment. The Schober test [12,13] was performed in all groups. Pain and mobility were also assessed 30 days after the completion of treatment.

### 2.3. Randomization

Randomization was conducted employing Random Allocation Software (version 1.0, 2010). The program generated allocation sequences automatically and provided concealed assignment until allocation, thereby minimizing the risk of selection bias. This method ensured that the randomization procedure was both reproducible and unbiased.

### 2.4. Statistical Analysis

The results of the study were subjected to statistical analysis, where the following methods were used: at the sample level, statistics that are estimators of the parameters of distribution of a random variable (e.g., arithmetic mean, sample median, sample standard deviation, sample asymmetry coefficient) and one-way analysis of variance (ANOVA). Hypothesis verification was performed adopting the commonly used threshold level of significance α = 0.05. If the test probability *p* obtained at the stage of the procedure used was lower than the adopted α (*p* < 0.05), the null hypothesis was rejected. Assuming a significance level of α = 0.05 and equal group allocation, a sample of 70 participants per group (n = 280) provides approximately 80% power to detect a medium effect size (Cohen’s f ≈ 0.30). Calculations were performed using the statistical package SPSS 20.0 for Windows. The results obtained are presented in tables and graphs.

## 3. Results

The results of this study were subjected to statistical analysis, where the following methods were applied: statistics were determined at the sample level, using estimators of the parameters of the distribution of the random variable (e.g., arithmetic mean, sample median, sample standard deviation, sample asymmetry coefficient). Sample distribution by treatment method, gender and age is presented in Table 1 and Table 2.

**Figure 1:**
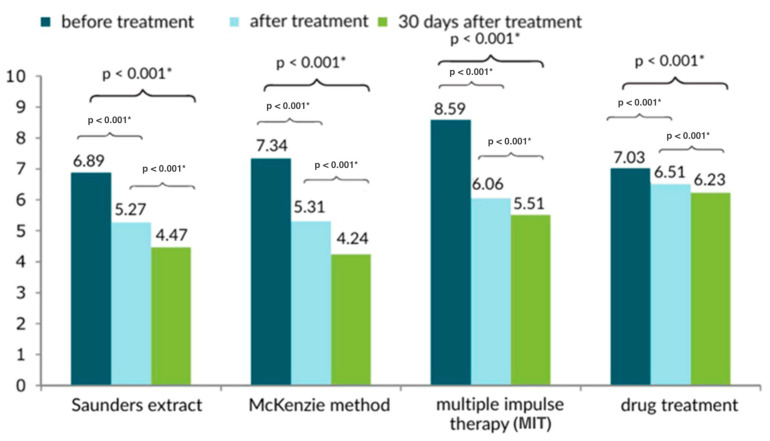
Comparison of the results of the subjective pain assessment of the lower back treatment method based on the Laitinen scale [0–16 points] in three measurements; * statistically significant.

**Figure 2:**
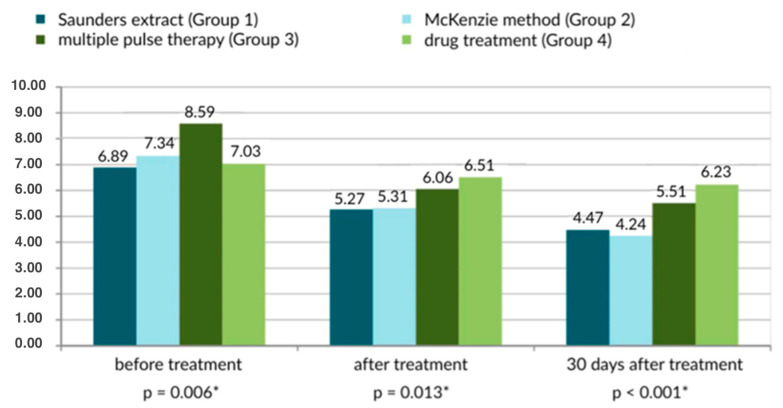
Subjective pain scores based on the Laititnen scale [0–16 points], according to the method of treatment of the lower spine. There were 70 participants in each analyzed group; * statistically significant.

On the Laitinen scale, the McKenzie method of therapy produced the best results (Figure 1 and Figure 2). The Saunders traction method was similarly effective.

**Figure 3:**
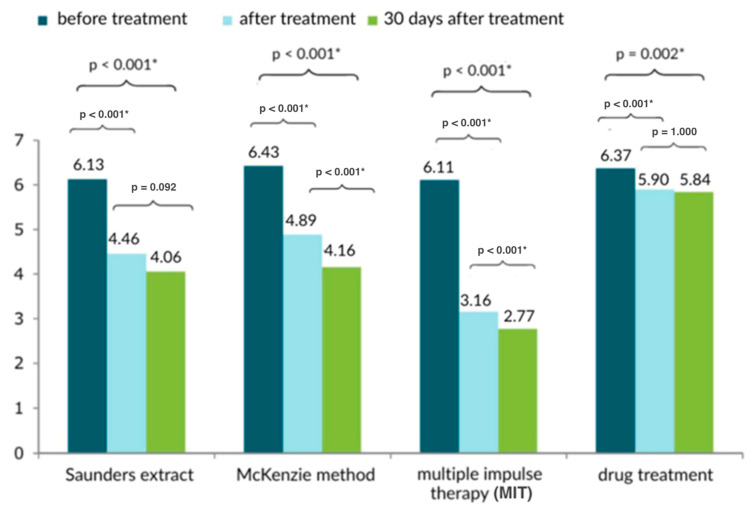
Comparison of the subjective pain scores of the lower back treatment method based on the VAS scale in three measurements; * statistically significant.

**Figure 4:**
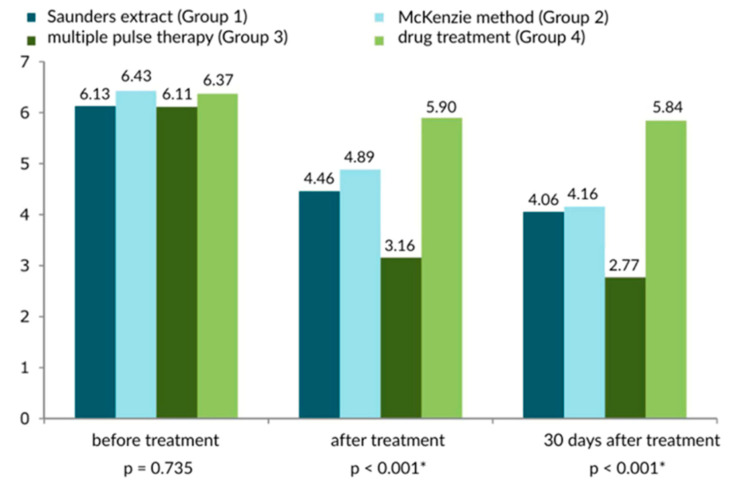
Results of the subjective pain assessment based on the VAS scale according to the lower back treatment method. There were 70 participants in each analyzed group; * statistically significant.

Figure 3 and Figure 4 present data on the evaluation of differences for post-treatment scores (immediately after and at 30 days after treatment) using each treatment method. In light of the test used (F-test of analysis of variance for post-treatment measurement and Welch’s test for measurement at 30 days after treatment), the MIT method yielded significantly different results on the VAS scale than the others (*p* < 0.05). However, all types of therapy used showed a significant improvement after treatment, as well as at 30 days after treatment completion.

In addition, in the statistical comparison between groups, the improvement was most significant in the MIT-treated group both after treatment and after 30 days.

**Figure 5:**
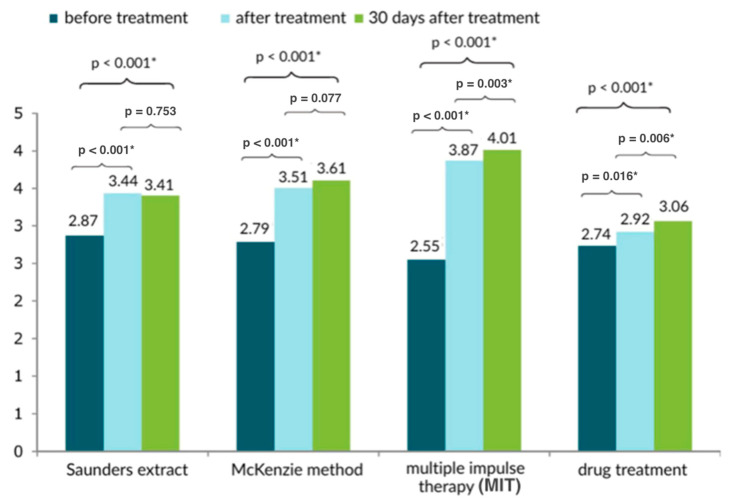
Comparison of mobility results and muscle tension of the treatment method of the lower spine based on the Schober test in three measurements. There were 70 participants in each analyzed group; * statistically significant.

**Figure 6:**
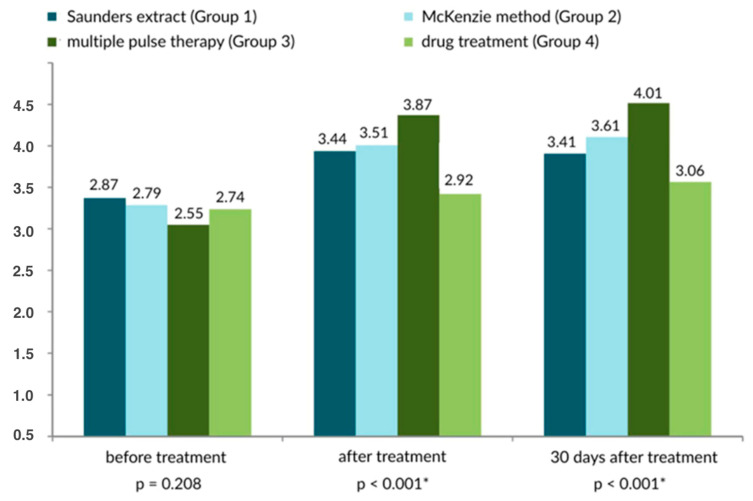
Results based on the Schober test according to the lower back treatment method. There were 70 participants in each analyzed group; * statistically significant.

Regarding the results of the Schöber test, as shown in Figure 5 and Figure 6, the multiple impulse method (Group 3) was highest (best), with slight—though significant—differences compared to the McKenzie method (Group 2). In contrast, the effects of MIT were significantly better compared to drug treatment (Group 4). At 30 days after treatment, the muscle tension rating was significantly lower, as evidenced by the trim greater range of motion of the spine in flexion.

## 4. Discussion

The analysis of the data presented in the figures clearly shows that the results of the Schöber test were highest (best) for the multiple impulse method (Group 3), with slight—though significant—differences compared to the McKenzie method (Group 2). In contrast, the effects of MIT were significantly better compared to drug treatment (Group 4). At 30 days after treatment, the muscle tension was significantly lower, as evidenced by a significantly greater range of motion of the spine in flexion.

Standards of care in clinical practice, linked to the International Classification of Functioning, Disability and Health, Academy of Orthopaedic Physiotherapy of the American Physiotherapy Association, include guided exercises of various types for both acute and chronic pain syndrome for this part of the spine. Another recommendation covers manual and other guided therapies for chronic lower back pain. Despite the general principles of applying these treatments, the approach of physiotherapists does not include the varied positions taken by physicians, psychologists, or ergonomists [14], as in the patient-centered therapy method [15].

Combining specialized pharmacological methods with rehabilitation seems to yield positive results. It is also worth paying attention to a three-stage concept of spine pathology treatment. These methods require further research in comparative or comprehensive therapies [16,17,18,19,20]. Physical therapy methods seem to be underestimated in physiotherapeutic recommendations [14], which show that laser therapy significantly reduces pain, and magnetic fields reduce muscle tension and increase mobility in LBP [21]. The combination of these methods has not been investigated in this context, although it seems to be beneficial; however, it should be noted that blue light (via a Sollux lamp) was used.

Goode A.P. et al. compared anatomical changes in traumatic studies with concentrations of markers of inflammation and pain: cytokines, proteoglycans, and neuropeptides. The pressure pain threshold (PPT), a marker of sensitivity to pressure-based pain, was measured. The concentration of baseline biomarkers was associated with time-dependent changes in lumbar spine structures (disc space narrowing (DSN) vs. osteophytes (OST)). Markers of inflammation and perceived pressure-based pain sensitivity were related with longitudinal worsening of LBP with DSN. Vertebral OST were not associated with pain of longitudinal origin [22].

The results indicate a correlation of multiple baseline biomarkers that are associated with the longitudinal deterioration of radiographic findings (Lumican, HA, and BDNF: DSN or OST) and the increased severity of LBP (CXCL6, HA, and PPT). Lumican has strong accuracy associated with degeneration images as a biochemical biomarker in further association with intervertebral disc degeneration; however, in OST, no association has been found. In the last few years, population-based studies have shown that DSN is associated with low back pain, and evidence suggests that it may be a risk factor for LBP. The current study does not focus on primary or secondary prevention of LBP resulting from degeneration of the spinal disc, but evidence suggests that exercise therapy in general—including stretching (e.g., the Saunders traction) and yoga—are good treatments for LBP [23], which is similar to the results in our study.

The various correlations of LBP, physical function, and radiographic features of the spine indicate the importance of the individual evaluation of the radiographic features and the measured outcomes. This study noted the importance of muscle load and tension for developing disc disease; so, this convergence was taken into account in the selection of the physical therapies. All the physical therapies in the presented study were based on mechanical action.

Single reports on the use of lumbar traction, the McKenzie method, and MIT confirm the analgesic efficacy of the methods used. In the study presented here, a comparison of these methods among themselves and the use of pharmacotherapy as a comparative therapy showed significant improvements in each of these treatments [3,4,5,9,10,11]. Nevertheless, the best effects were described for MIT when comparing groups, which can be explained by its strongest effect on muscle tone [5,24].

The assessment of the analgesic effect should also include patient conditions such as their psychological state during therapy and their sociocultural conditions [15,25]. Risk factors associated with lower back pain include older age, female gender, higher BMI, lower socioeconomic status, smoking, occupational factors, psychological stress, depression, sleep deprivation, fatigue, and pain in other parts of the body [25,26]. However, the analgesic results obtained generally correlate with a reduction in muscle tension, suggesting that the two are related and can be used to assess the effectiveness of treatment.

A meta-analysis suggested that future physiotherapy should be AI-assisted, as it may be more effective in relieving pain than conventional physiotherapy, improving both daily functional abilities and improving mental health in patients with NSLBP, including symptoms of depression and anxiety related with chronic pain. By offering personalized treatment plans and providing real-time feedback, AI-assisted physiotherapy is more tailored to the individual patient’s needs, potentially improving patient satisfaction and overall treatment outcomes [27,28,29], which is supported by previous reports of personalized treatment [15].

More and more often, doctors are considering regenerative therapy, which is yielding increasingly better long-term results [30].

Minimally invasive interventional methods—such as intervertebral joint or nerve blocks, epidural steroids, and radiofrequency ablation—usually provide insufficient or only short-term benefits at best. Even when somewhat effective, these procedures often have diminishing benefits and rarely result in significant lasting functional improvements. Repair neurostimulation has been proposed as a specific therapy for the treatment of CLBP in cases of multifidus muscle dysfunction. In this case, pharmacological treatment produced poorer results [30].

The regenerative method for LBP aims to restore stability, increase strength, and improve neuromuscular control. This has a positive effect on pain reduction (including discogenic pain) [31].

Implementation of the McKenzie method, ultrasound therapy, sensory–motor training, and Swiss ball exercises, alongside other therapeutic approaches, effectively alleviated pain and enhanced strength, balance, and the ability to carry out daily tasks [32]. A corrective approach has a positive impact on recovery rates, especially when classic physiotherapy methods are combined with new intervention techniques. The use of appropriately selected physiotherapy in LBP remains a challenge for modern rehabilitation.

The limitations of this study may include the lack of another study group in which all physical factors were assessed together in comparison to those used separately, although this would be a reason to take contraindications into account. Additionally, studies with a longer follow-up period (6–12 months), functional assessments, and patients outside the studied age range (30–60 years) are necessary.

## 5. Conclusions

Multiple impulse therapy functions as a valuable modality for pain control for treating patients with discogenic low back pain compared to McKenzie MDT and Saunders traction. MIT is well-tolerated by patients, completely safe, and non-invasive. Physiokinetic methods such as Saunders’ traction, McKenzie, and MIT showed greater analgesic efficacy when compared to drug treatment in patients with discogenic low back pain.

## Figures and Tables

**Table 1:** Sample distribution by treatment method and gender. The chi-square test of independence was used; *p* = 0.808.

Description		Patients Undergoing Treatment with Saunders Traction (Group 1)	Patients Undergoing McKenzie Treatment (Group 2)	Patients Undergoing Multiple Impulse Therapy (Group 3)	Patients Undergoing Pharmacological Treatment (Group 4)	Total
Women	n	35	36	32	31	134
	%	50.0%	51.4%	45.7%	44.3%	47.9%
Men	n	35	34	38	39	146
	%	50.0%	48.6%	54.3%	55.7%	52.1%
Total	n	70	70	70	70	280
	%	100.0%	100.0%	100.0%	100.0%	100.0%

**Table 2:** Age distribution statistics by treatment method.

Description	Patients Undergoing Treatment with Saunders Traction (Group 1)	Patients Undergoing McKenzie Treatment (Group 2)	Patients Undergoing Multiple Impulse Therapy (Group 3)	Patients Undergoing Pharmacological Treatment (Group 4)
Number of respondents	70	70	70	70
Average	45.40	45.16	44.41	45.84
5% trimmed mean	45.42	45.14	44.35	45.89
Median	45.00	44.00	43.00	47.00
Standard deviation	9.02	9.40	9.17	8.56
Minimum	30	30	30	30
Maximum	60	60	60	60
Range	30	30	30	30
Coefficient of asymmetry	0.101	0.142	0.148	−0.109
Kurtosis	−1.121	−1.275	−1.056	−0.956
Assessment of the significance of differences (ANOVA)	Levene’s test of homogeneity of variance: *p* = 0.662 ANOVA: *p* = 0.821			

## Data Availability

The data are available from the corresponding author if required.
